# Facts and Observations on Diabetes

**Published:** 1817-03

**Authors:** C. W. Smerdon


*For the London Medical and Physical Journal.*

*Facts and Observations on Diabetes;* by Mr. C. W. SMERDON.

MRS. C. a native of Ireland, and mother of many children, between thirty and forty years of age, was attacked, about fifteen months ago, with raging thirst, violent spasmodic pain about the scrobiculus cordis, occasional sickness and vomiting, and severe pain of the loins. Her drink she estimated at from ten to twelve quarts a day; and her urine, which was always colourless and insipid, was in proportion to the quantity of liquid taken in. She rarely perspired even in health, unless in consequence of considerable exertion; but she is now sensible of constantly dry and arid skin. At the time of this attack, she was a nurse, and, notwithstanding the violence of the disease, her milk continued undiminished, and, as far as she knew, its quality was the same. Her appetite has never been craving. A continual burning heat of her feet and hands was particularly distressing to her, from which she found some alleviation by keeping them exposed to a stream of cold air. She has constantly an unpleasant feeling of heat extending down the œsophagus into the stomach, but the violent pain of this viscus and the loins, attended with sickness, usually recurred in paroxysms after short intervals. The severity of these attacks was so great as to confine her wholly to bed, and all the other symptoms of her disorder were likewise greatly increased. She remarked, that these paroxysms were always preceded by an increase of thirst and heat of skin; and she is convinced that they were either produced, or greatly aggravated, by the immense quantity of cold liquids which her constant craving for drink compelled her to take in. On one occasion, so great was the pain of the bowels and loins, that the medical man first called in, being a stranger to the existing disease, applied a blister to the abdomen, under the idea that she had enteritis. This attack was alleviated by laudanum and purgatives. Before she was the subject of diabetes, her spirits were remarkably good, and her constitution apparently robust; but, without any secret cause, she afterwards became low, careless of her domestic domestic concerns, and at times so averse to amusements, that she has sat a whole evening with a chearful party of her most intimate friends without speaking a word, or taking any notice of surrounding objects.

In consultation, it was determined to give blood-letting a fair trial, but the plan was desisted from after the first bleeding, because the blood drawn had a perfectly healthy appearance. The warm-bath always increased the diabetic symptoms, particularly the heat of her hands and feet. Blisters were applied to her loins, and kept open for some time without any beneficial effect; and, with no better success, she took the carbonated lime water, alum whey, and emetics. At length, the failure of all medical assistance in her own country, and the alarming state of her health, determined her friends to bring her to England. She went to Shrewsbury, and consulted Dr. Darwin, who recommended a diet consisting entirely of animal food. This plan, which she persisted in with great difficulty for about a week, completely disagreed with her. She became much worse, and, notwithstanding her weakness was great, she was recommended to lose no time in coming to Clifton. For the first two days after her arrival here, her thirst was extreme, and she estimated her drink at two gallons a day; from this time she began apparently to recover; the craving for drink became more moderate; and her strength and spirits returned in proportion to the decrease of the disease. When the urine evacuated was decreased to seven pints a day, I obtained some of it to examine. It was clear, depositing no sediment on standing, and hardly in appearance distinguishable from common water; its smell was faintly urinous; taste rather salt; but, as it regards sweetness, perfectly insipid. The oxymuriate of mercury, and the muriate of lime, threw down precipitates. On standing for two days, it began to exhale a fœtid odour, and floculi were deposited. Eight ounces, by slow evaporation, yielded twenty-five grains of solid matter; from which I obtained four grains of crystallized urea, seventeen grains of saline matter soluble in water, and four grains of a residuum insoluble in either water or spirit of wine. Hence this urine deviated, in its component parts, from healthy urine, only by containing a greater proportion of water.*

Mrs.

* In the Medico-Chirurgical Transactions for 1812, there is a case of diabetes insipidus, published by Dr. Bostock, in which the residuum, left by slow evaporation of the urine, did not differ in its physical

Mrs. C. has been here now about six months; at the end of the second and fourth month she was weighed, by which it was ascertained that she had gained two pounds and a half within that time; her appearance, strength, and spirits, are also much improved. From the commencement of her disease to the period that she left Ireland for this country, she is convinced that she must have lost in weight more than twenty pounds. A more convincing proof than this cannot be, of the decided check which has been given to the disease, either by the powers of nature, the change of air, the Hotwell water (which she drinks medicinally), or the remedies employed;* but, although all the symptoms are greatly mitigated, it does not appear that she has entirely lost any one of them: she still feels the heat of her stomach and œsophagus; her tongue is furred and chapped; her skin dry and glossy; and she is seldom without a sense of uneasiness in her loins,—this rarely amounted to pain, but is a feeling of

---

physical properties from the extract of healthy urine; this corresponds exactly with the case above narrated. But, on chemical examination, the doctor found, that this urine contained a great quantity of animal matter; so that, while his patient was passing daily seven pounds troy of urine more than the average quantity, she was discharging in that time between nine and ten ounces of solid matter, or eight ounces and a half more than the regular quantity. Without seeking for any other cause, we have here a very sufficient one to account for the gradual waste of the body in this disease; and not only in this disease, but also in others, in which, notwithstanding the appetite for food is unimpaired, the subjects of them die in a state of the greatest emaciation. In tabes mesenterica and phthysis pulmonalis, which are frequently attended by a depraved appetite for food, the excreta will, I believe, pretty generally be found loaded with nutritive or animal matter. The average quantity of solid matter daily lost by urine, according to Dr. Bostock, is six drams. Wilson, in eight pints of urine, daily discharged above five ounces of solid matter; and Mr. W. whilst at Clifton, averaged about three ounces. In many of the cases on record, if we may judge by the bulk of urine evacuated, (which, however, is not a just criterion,) the quantity of solid matter lost must have been a great deal more than either of the above. But this subject, in the generality of cases published, has not been attended to as much as it ought. Mrs. C.'s case is a remarkable instance of the reverse of what usually takes place, and particularly with respect to the case of Dr. Bostock: in six pints of urine (which is just double the daily average quantity), Mrs. C. only lost four drams and a half of solid matter.

* These were, a blue pill taken every alternate night, and a little sulphur in the morning.

weakness, weakness, as if she was falling away from herself as she expresses it ;† her pulse is low, and ranges from sixty to sixty-four in the minute. Her appetite is good, and, until lately, she has eaten a large proportion of animal food. Her thirst has not been urgent; she has drunk, for some time past, seven pints of the Hotwell water in the twenty-four hours, a part of which, however, rather as a medicine than for the mere purpose of allaying thirst. About three weeks ago, she commenced taking lime water in divided quantities of twelve ounces a day ; and, at the same time, changed her diet for one principally consisting of vegetable and farinaceous food. She has also reduced the quantity of drink as much as possible by these means, as might very naturally be inferred; the diabetic symptoms are still further diminished, particularly the fever, the heat of her stomach, and the unpleasant sensation of her loins.

Such are the general, though imperfect, outlines of this interesting case ; she has taken but little medicine since she has been here, except what has already been noticed ; for, what remedy could conscientiously be recommended for an obscure case like the present, in which the symptoms are so greatly mitigated without any?

It would be important in a pathological view to ascertain, whether the diabetes insipidus be a distinct disease, or merely a variety of the sweet kind. Now, if we contrast the symptoms of each, we shall be so struck with their similarity as to be satisfied that they arise from one and the same cause ; but, if we merely examine the urine of each species without attending to symptoms, the conviction on our minds would be quite the reverse. Before, however, we could form a correct judgment on this subject, it would be necessary to determine the following queries.

1st, Do the peculiarities in the urine of the saccharine and insipid diabetes vary, or alternate with each other?

2dly, Does the presence or absence of saccharine matter constitute the only distinctions between insipid and sweet urine.

From several examinations, I am satisfied, that Mrs. C.'s urine has not varied from the analysis I have just given since her residence here ; and, I have good authority for believing, that it never has, further than that of containing a greater

† It is worthy of remark, that this sensation of debility is greater at night when in bed, and is sometimes accompanied by an unaccountable lowness of spirits, which either disturbs her rest, or awakes her out of sleep.

proportion proportion of solid matter. If, therefore, a conclusion might be drawn from a single case, the first question must be answered in the negative. But, in the case before alluded to, Dr. Bostock, on one occasion, detected saccharine matter, although he found in the same specimen all the constituent properties of the urine: he was, therefore, led to conclude, that the insipid is sometimes converted into the saccharine diabetes, and that the two states probably alternate with each other, until, as the constitution becomes more and more impaired, the saccharine state of the urine predominates. This opinion, I believe, will not bear the test of experience, but the high authority from whence it originated is alone sufficient to render it worthy of notice.

If, however, there be one well marked case of diabetes insipidus, which, from its commencement and for a long period afterwards, has uniformly retained its characteristic marks, such a case, even on this account, is worthy of consideration, since it undoubtedly proves, that the morbid actions, on which the altered state of the urine depends in the saccharine diabetes are not to be found in every case of the insipid species. This distinctive mark, if established, must also lead to some distinction in practice. The animal diet is only intended to prevent the formation of sugar, by abstracting that portion of the food which is susceptible of the fermentative process; but surely, where existing circumstances do not, on this principle, at all indicate the necessity of such a diet, its adoption must evidently be the means of exasperating all the worst symptoms of the disease.

The second question may be answered with much greater certainty. The urine of the saccharine diabetes, when evaporated, leaves a black treacle-like residuum, and which happens whether it contains sugar or not, as we learn by Mr. W.'s case. This morbid appearance of urine is occasioned by extraneous matter, and is, I believe, peculiar to the diabetes mellitus. The absence of urea, and the effervescent quality of the urine, when evacuated, also characterize the disease to which they belong; and point out the certainty of some established distinction between it and the insipid diabetes; in which, neither these peculiarities, nor others which have been mentioned, are found.

It may, however, very justly be doubted, if the establishment of these points can be of any utility to us in practice; and, indeed, from a review of all the symptoms of this case, I am decidedly of opinion, that it is as favourable for the bleeding plan, as any that has ever been published.

*Clifton, Bristol; Oct.* 27, 1816.

